# Evolving Demographics and Outcomes in Surgically Treated Acute Type A Aortic Dissection: A Fifteen-Year Regional Experience

**DOI:** 10.3390/medicina61122236

**Published:** 2025-12-18

**Authors:** Elisa Mikus, Mariafrancesca Fiorentino, Diego Sangiorgi, Antonino Costantino, Simone Calvi, Elena Tenti, Anna Milione, Sara Valota, Alberto Tripodi, Carlo Savini

**Affiliations:** 1 Cardiovascular Department, Maria Cecilia Hospital, GVM Care & Research, 48033 Cotignola, Italy; 2 Division of Cardiac Surgery, Department of Advanced Biomedical Sciences, University of Naples “Federico II”, 80138 Naples, Italy; 3 Department of Biomedical and Neuromotor Sciences (DIBINEM), University of Bologna, 40127 Bologna, Italy; 4 Department of Experimental Diagnostic and Surgical Medicine (DIMEC), University of Bologna, 40127 Bologna, Italy

**Keywords:** acute type A aortic dissection, regional epidemiological analyses, gender incidence and outcomes

## Abstract

*Background and Objectives*: Acute type A aortic dissection (ATAAD) remains a life-threatening condition requiring prompt surgical management. Over the last decades, improvements in diagnosis, surgical techniques, and perioperative care have influenced patient characteristics and outcomes. This study analyzes temporal trends in the clinical profiles and results of patients surgically treated for acute type A aortic dissection (ATAAD) in a Northern Italian region over a fifteen-year period. *Materials and Methods*: All consecutive patients undergoing emergency surgery for acute Stanford type A aortic dissection or acute intramural hematoma (IMH) between January 2010 and December 2024 were retrospectively reviewed. Patients with chronic penetrating atherosclerotic ulcer or traumatic etiology were excluded. Demographic, clinical, and perioperative variables were analyzed to assess temporal changes. Trends were evaluated using linear regression and Cochran–Armitage tests for trend. *Results*: A total of 427 patients underwent surgery for AAD during the study period. The proportion of patients presenting with preoperative intubation significantly decreased over time (*p* for trend < 0.05), as did the incidence of preoperative shock (*p* for trend < 0.001). Conversely, the mean EuroSCORE showed a non-significant increase over the years. No significant differences were observed in age or other baseline parameters. A non-significant but progressive increase in female representation was observed over time (*p* = 0.064). Given this observation, a sex-based subanalysis was performed: women were significantly older (*p* < 0.001) and presented with higher EuroSCORE values (*p* < 0.001) compared to men, yet postoperative mortality was similar between sexes. This finding contrasts with recent reports suggesting worse outcomes among female patients. *Conclusions*: Over fifteen years, patients undergoing surgery for acute type A aortic dissection have shown decreasing rates of preoperative critical conditions, reflecting earlier diagnosis and improved management. Despite higher operative risk scores, women demonstrated comparable short-term survival to men within our regional program. Multivariable analysis showed that sex was dependently associated with in-hospital mortality.

## 1. Introduction

Acute type A aortic dissection (ATAAD) encompasses a spectrum of catastrophic conditions of the thoracic aorta, primarily including acute aortic dissection and intramural hematoma (IMH). Despite its relatively low incidence—estimated at approximately 2–4 cases per 100,000 persons per year—ATAAD is associated with extremely high morbidity and mortality, particularly in the absence of prompt diagnosis and timely surgical or endovascular intervention [1,2,3]. Current international guidelines emphasize the importance of rapid recognition and immediate surgical referral in order to improve survival outcomes [1,3]. However, while data from large national registries and multicenter studies have contributed to a broader understanding of the disease, regional analyses remain comparatively scarce. Over the past two decades, significant contributions have been made by large-scale collaborative efforts such as the International Registry of Acute Aortic Dissection (IRAD). IRAD data indicate that patients with type A aortic dissection present at a mean age of 62 years, with a male predominance of approximately two-thirds [4,5]. Operative mortality—although markedly improved over time—remains around 18–25% in most series [3,6]. European registry studies have confirmed these findings [1], reporting progressive increases in age at presentation and improved early survival, largely attributable to advances in surgical techniques and perioperative management. Nonetheless, differences in healthcare organization, referral systems, and population demographics can significantly influence presentation and outcomes, suggesting that international data may not fully capture regional realities.

In this context, regional epidemiological analyses remain essential to complement multicenter evidence. Local data allow for the identification of specific trends, evaluation of referral system performance, and a more accurate understanding of real-world surgical outcomes. A mid-sized Northern Italian region with a centralized referral system and consolidated expertise in complex aortic surgery represents an ideal setting to explore these aspects.

The present study aims to evaluate temporal trends, demographic characteristics, perioperative outcomes, and seasonal variation in a large cohort of patients undergoing surgery for acute type A aortic dissection and acute intramural hematoma of the ascending aorta, treated as part of the same clinical spectrum (Acute Aortic Syndrome). The specific objectives of the study are: (i) to describe changes over time in patient demographics, clinical presentation, and surgical techniques; (ii) to assess outcomes using multivariable adjustment to identify independent predictors of mortality; and (iii) to explore sex-related differences in presentation, management, and short-term outcomes. Special attention was given to sex-related differences, as recent evidence has highlighted the potential prognostic impact of gender in acute type A aortic dissection, though findings have been inconsistent across studies. By evaluating a 15-year single-center experience within a regional referral network, we aim to provide insights into evolving patient characteristics, surgical strategies, and outcomes in this surgically treated cohort.

## 2. Materials and Methods

### 2.1. Study Design and Population

This is a retrospective, observational study including all consecutive patients undergoing emergency surgery for acute type A aortic dissection (ATAAD) or acute intramural hematoma of the ascending aorta between January 2010 and December 2024. ATAAD was defined as acute Stanford type A aortic dissection or intramural hematoma requiring open surgical intervention, in accordance with current international guidelines. Patients presenting with penetrating atherosclerotic ulcer (PAU) or traumatic aortic injury were excluded. The diagnostic approach for suspected acute aortic syndrome was consistent over the study period and based on thoraco-abdominal contrast-enhanced CT angiography, which represented the institutional and regional gold standard. No relevant changes in imaging modalities, acquisition protocols, or diagnostic workflow occurred over time. Intramural hematoma was defined radiologically as focal or circumferential thickening of the aortic wall without evidence of intimal flap, in accordance with ESC guidelines [1]. Only surgically treated intramural hematoma cases were included in the analysis.

A total of 427 patients were included in the analysis. For temporal assessment, patients were stratified into five consecutive 3-year periods as follows:

- Period 1: 2010–2012 (*n* = 75)
- Period 2: 2013–2015 (*n* = 72)
- Period 3: 2016–2018 (*n* = 104)
- Period 4: 2019–2021 (*n* = 72)
- Period 5: 2022–2024 (*n* = 104)

This time-based stratification allowed for the evaluation of evolving trends in patient demographics, preoperative presentation, and outcomes over the fifteen-year study period. Patient referral followed a standardized regional emergency network system, which identified our institution as the hub center for ATAAD/IMH for all emergency departments and cardiology services across the Romagna provinces. This organizational model ensured a stable pathway for patient transfer and homogeneous case selection throughout the study period.

To improve reproducibility, the following definitions were applied uniformly:

- Hemodynamic instability: systolic blood pressure < 90 mmHg or the need for continuous vasoactive support to maintain SBP ≥ 90 mmHg.
- Shock: hemodynamic instability accompanied by signs of hypoperfusion, including altered mental status, oliguria < 0.5 mL/kg/h, or serum lactate > 2 mmol/L.
- Neurological deficit: new focal neurological impairment at presentation or transient loss of consciousness attributable to suspected cerebral malperfusion.
- Respiratory failure requiring intubation: requirement for invasive mechanical ventilation prior to surgical induction due to hypoxemia (SpO_2_ < 90% despite supplemental O_2_), airway compromise, or reduced consciousness.

### 2.2. Data Collection

Demographic and clinical data were extracted from electronic medical records and operative notes. Variables included age, sex, body mass index, comorbidities (hypertension, diabetes mellitus, coronary artery disease, chronic kidney disease, prior cardiac surgery), and Stanford classification. Preoperative clinical parameters included hemodynamic status at presentation (stable, shock), requirement for preoperative intubation, and calculated EuroSCORE II and Logistic EuroSCORE. Operative variables encompassed the type of surgical repair (ascending aorta replacement, hemiarch, total arch, or root replacement), cardiopulmonary bypass and cross-clamp times and adjunctive procedures. Postoperative outcomes included in-hospital mortality, stroke, myocardial infarction, acute kidney injury requiring dialysis, re-exploration for bleeding, duration of intensive care unit (ICU) stay, and total hospital stay.

All data were independently reviewed by two investigators to ensure accuracy and consistency.

### 2.3. Statistical Analysis

After Shapiro–Wilk test, continuous variables were expressed as median (interquartile range, IQR) and compared using the Kruskal–Wallis test. Categorical variables were expressed as counts and percentages and compared using the Chi-square or Fisher’s exact test. Temporal trends across the years were analyzed using Cuzick test for continuous variables or Cochran–Armitage test for trends for categorical variables. A *p*-value < 0.05 was considered statistically significant. Comparative analyses between male and female patients were performed to explore three-year periods and sex-related differences in demographics, preoperative risk profile, and outcomes; the three-year periods. The study period was divided into five three-yearly intervals to ensure an adequate number of patients per interval and to capture temporal trends without excessive data fragmentation. To identify independent predictors of mortality, a multivariable logistic regression model was implemented, including age, sex, preoperative shock, intubation status, intramural hematoma, logistic EuroSCORE, arch replacement, cardiopulmonary bypass (CPB) time, and year of surgery. To minimize overfitting, variable selection was performed using the LASSO method with 10-fold cross-validation to identify the most informative predictors. The same approach was implemented to assess whether sex was independently associated with arch replacement. For this model, only preoperative variables were considered (CPB time was excluded), while Area Under the Curve for model discrimination and Hosmer and Lemeshow test for calibration were reported. Before performing the multivariable models, missing data were imputed using random forest imputation for categorical variables and predictive mean matching for continuous variables. The imputation was performed with 100 trees and 5 iterations, using predictive mean matching with k = 3 to ensure realistic values for continuous variables; complete cases were 408/427 (96%). Seasonal variation was assessed using a negative binomial regression model for proportions, with seasons defined by calendar months (spring: Mar–May; summer: Jun–Aug; autumn: Sep–Nov; winter: Dec–Feb). Rates were expressed as percentages of total cases per period. All analyses were performed with R 4.5.0 (R Foundation for Statistical Computing, Vienna, Austria).

## 3. Results

### 3.1. Study Population

**Figure 1:**
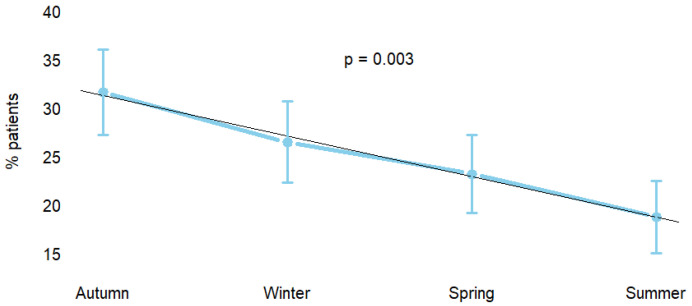
Seasonal variation in acute type A aortic dissection.

Between January 2010 and December 2024, a total of 427 patients underwent surgical repair for acute aortic dissection in our region. The distribution of surgical interventions was also analyzed in relation to the season in which the acute aortic syndromes occurred. The analysis revealed that the highest proportion of cases was observed during the autumn months (*n* = 135, 31.6%), followed by the winter season (*n* = 113, 26.5%). Conversely, a lower frequency was recorded during spring (*n* = 99, 23.2%) and summer (*n* = 80, 18.7%). This seasonal variation demonstrated a statistically significant trend (*p* = 0.003), suggesting a potential influence of environmental or climatic factors (Figure 1). A sensitivity analysis restricted to the COVID-19 period (2019–2021) confirmed the same seasonal pattern (*p* < 0.001).

**Figure 2:**
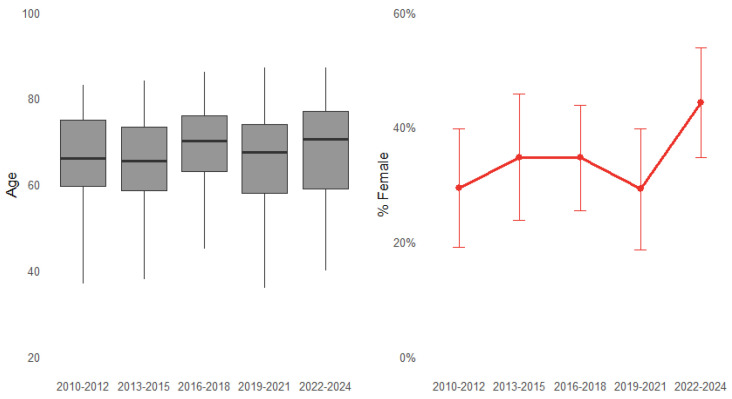
Demographic characteristics according to three-year periods.

The median age of the cohort was 68 years (IQR 59–76), with 65% males (*n* = 277) and 35% females (*n* = 150). Across the five study periods, no statistically significant differences were observed in patients’ age or major comorbidities (*p* for trend = NS). Nevertheless, a non-significant upward trend in age was noted (*p* = 0.064), indicating that patients undergoing surgery in recent years were generally older. A progressive increase in the proportion of female patients was also observed (*p* for trend = 0.074), rising from 29.3% in the earliest period to 44.2% in the most recent triennium (Figure 2).

**Figure 3:**
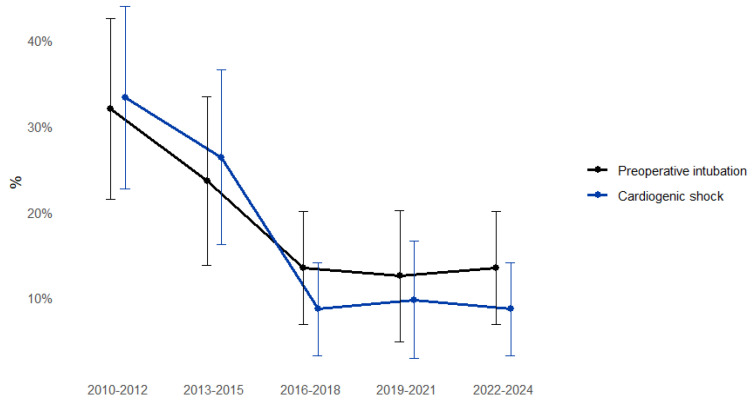
Clinical characteristics according to three-year periods.

The incidence of preoperative intubation significantly decreased over time (*p* for trend < 0.05), as did the proportion of patients presenting in cardiogenic shock (*p* for trend < 0.001), suggesting that patients are increasingly reaching surgery in more stable clinical conditions (Figure 3).

However, this improvement was not associated with a corresponding reduction in predicted operative risk, as the EuroSCORE II remained relatively high and showed a gradual, though non-significant, increase across study periods (*p* for trend = NS). In addition, a progressive increase in the proportion of patients presenting with preoperative neurological deficits was observed over time (12.5% in the last triennium), indicating a higher percentage of patients reaching surgery with evidence of cerebral malperfusion or stroke symptoms at admission (Table 1).

### 3.2. Operative Characteristics

Regarding the type of surgical procedure, most technical aspects remained consistent over time. The use of axillary artery cannulation showed no significant variation across study periods (*p* for trend = 0.505) and continued to be the preferred arterial access in the majority of patients (over 60%). Aortic cross-clamp times remained relatively stable throughout the study, whereas cardiopulmonary bypass (CPB) durations increased significantly over time (*p* for trend < 0.001). This trend paralleled a progressive rise in the proportion of patients undergoing total aortic arch replacement (*p* for trend = 0.002), which may account for the increase in CPB time but not in cross-clamp duration. The rate of aortic root replacement remained stable during the study period (*p* for trend = NS). All patients undergoing total arch replacement were managed with systemic circulatory arrest, and in every case cerebral protection was provided through antegrade cerebral perfusion under moderate hypothermia (26 °C). (Table 2 presents operative details by study period.)

### 3.3. Outcomes and Mortality

The overall in-hospital mortality for the entire cohort was 10.8%, with no statistically significant difference observed among the five 3-year study periods (*p* for trend = 0.572). Similarly, the incidence of acute kidney injury (with or without the need for dialysis), re-exploration for bleeding, stroke and postoperative atrial fibrillation remained stable over time (*p* for trend = NS). Conversely, a statistically significant reduction in blood and blood-product transfusions was observed throughout the study period (Table 3).

**Figure 4:**
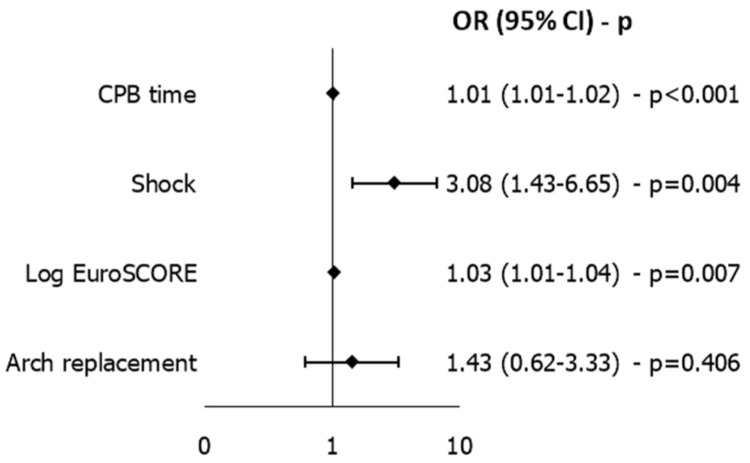
Independent Predictors of Mortality Identified by LASSO-Selected Multivariable Logistic Regression. CPB time is expressed in minutes. AUC = 0.814.

Independent risk factors for mortality were cardiopulmonary bypass time [OR 1.011 (95% CI 1.007–1.016), *p* < 0.001], shock [OR 3.078 (95% CI 1.425–6.648), *p* = 0.004] and logistic EuroSCORE [OR 1.025 (95% CI 1.007–1.044), *p* = 0.007]. The model presented an AUC of 0.814 and a Hosmer–Lemeshow test *p*-value of 0.562 (Figure 4). Since 46 deaths were observed, the model is unlikely to overfit, especially considering that it selects only four predictor variables; a sensitivity analysis on complete cases was performed, yielding comparable results.

### 3.4. Sex-Based Analysis

When comparing sexes in surgically treated patients, female patients were significantly older than males (*p* < 0.001) and presented with a higher EuroSCORE II (*p* < 0.001). The prevalence of preoperative shock and intubation was comparable between sexes (*p* = NS for both). Other preoperative characteristics were also similar, except that female patients exhibited a lower body mass index and better renal function, as reflected by lower preoperative serum creatinine levels (*p* < 0.001 for both). Notably, intramural hematoma was significantly higher among women compared with men (*p* = 0.020) (Table 4).

**Figure 5:**
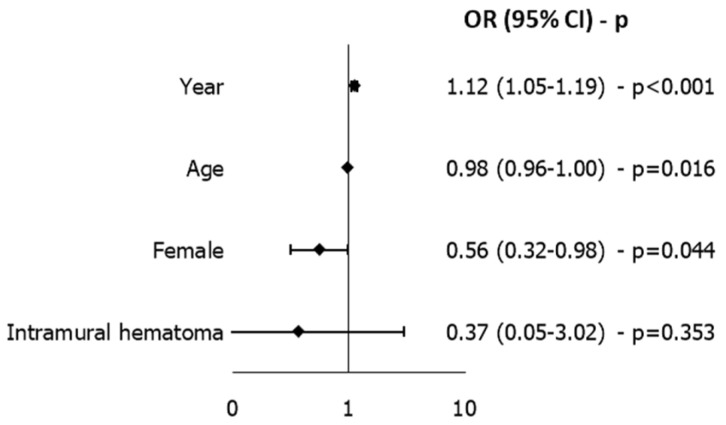
Independent Predictors of Arch Replacement Identified by LASSO-Selected Multivariable Logistic Regression. AUC = 0.673.

Operative strategies were generally comparable between sexes; however, female patients had significantly shorter aortic cross-clamp times (*p* < 0.001), and were less frequently subjected to total aortic arch replacement (*p* = 0.009), as confirmed by the multivariable model (Figure 5). Despite this, women required blood transfusions more often than men (*p* = 0.013). Postoperative outcomes were overall similar between sexes, with no statistically significant differences observed in major complications such as stroke, re-exploration for bleeding, or atrial fibrillation. The incidence of acute kidney injury and dialysis was, however, higher in male patients, consistent with a worse preoperative renal function. Importantly, in-hospital mortality was identical in both sexes (11%; *p* = 1.000), indicating comparable short-term survival outcomes despite the higher preoperative EuroSCORE II observed in female patients (Table 5 summarizes sex-based intraoperative characteristics and postoperative outcomes).

Multivariable analysis identified year as the only independent risk factor for arch replacement [OR 1.116 (95% CI 1.052–1.185), *p* < 0.001], whereas female sex [OR 0.557 (95% CI 0.315–0.984), *p* = 0.044] and age [OR 0.975 (95% CI 0.955–0.995), *p* = 0.016] were found to be protective factors. The model presented an AUC of 0.673 and a Hosmer–Lemeshow test *p*-value of 0.795 (Figure 5); since 90 arch replacements were performed, the model is unlikely to overfit, especially considering that it selects only four predictor variables.

## 4. Discussion

### 4.1. Temporal Trends in Type A Acute Aortic Dissection Surgery over Fifteen Years

This fifteen-year single-center analysis provides a comprehensive overview of temporal trends in the presentation, management, and outcomes of patients undergoing surgery for type A acute aortic dissection (ATAAD) within a regional referral network encompassing the entire Romagna area of Northern Italy. To our knowledge, this represents one of the few regional experiences spanning more than a decade of uninterrupted surgical activity conducted by a dedicated team under standardized operative and perioperative protocols. Such a setting provides a unique framework to assess how patient characteristics, surgical strategies, and outcomes have evolved in a stable and well-organized healthcare environment. The overall temporal trends in surgical case volume of surgically treated ATAAD remained relatively stable throughout the study period, suggesting that the regional burden of this life-threatening condition has not substantially changed despite progressive population aging and increased diagnostic awareness. In the Romagna region, the estimated incidence was approximately 2–4 cases per 100,000 persons per year, consistent with previously reported European data [1]. Since our center serves as the referral hospital for the entire Romagna area, this incidence was calculated based on the number of patients who underwent surgery at our institution over the total population of the Romagna region. The number of operated patients showed modest fluctuations across the five three-year periods. This temporal pattern may reflect broader healthcare dynamics, particularly the impact of the COVID-19 pandemic during 2019–2021, which likely contributed to the transient reduction in surgical volume due to limited hospital accessibility and delayed presentation, as many patients avoided medical evaluation despite symptoms. The seasonal variation observed, with a peak incidence during autumn and winter months, may reflect multiple environmental and physiological influences. Colder temperatures have been associated with increased sympathetic activity, higher systemic blood pressure, and greater mechanical stress on the aortic wall, potentially precipitating acute events. Seasonal fluctuations in physical activity, incidence of respiratory infections, and inflammatory responses may further contribute to susceptibility. While speculative, these factors provide plausible mechanisms for the observed pattern, which was consistent even during the COVID-19 period. Future studies incorporating population-level incidence estimates, emergency admission data, or broader regional registries will be necessary to determine whether a true seasonal variation exists and to clarify the potential drivers underlying these trends.

Although the observed increase in patient age over time did not reach statistical significance (*p* for trend = 0.064), a clear upward trend was evident, suggesting that progressively older patients are now being referred for surgical repair. Notably, patients in recent years tended to reach surgery in better preoperative condition, as evidenced by a significant decline in the incidence of cardiogenic shock and preoperative intubation. This improvement likely reflects advances in early diagnosis, the implementation of efficient referral pathways, and optimization of perioperative management. These findings parallel the trends described by the International Registry of Acute Aortic Dissection (IRAD), which reported a progressive decline in preoperative hemodynamic instability and an associated improvement in surgical outcomes [3,4]. Although the proportion of patients presenting with preoperative shock and intubation decreased over time, predicted operative risk, as reflected by EuroSCORE II, slightly increased. This apparent paradox can be explained by multiple interrelated factors. First, the cohort has progressively aged, with older patients inherently contributing to higher risk scores, as age is a major determinant of operative mortality in validated risk models. Second, the accumulation of comorbidities and female sex, which is associated with higher baseline surgical risk in acute aortic dissection [5], became more represented in later years, also contributing to higher EuroSCORE II values. Third, evolving referral pathways and the optimization of the regional aortic network may have enabled higher-risk patients, previously deemed inoperable, to reach our center for emergency surgery. Moreover, subtle changes in preoperative presentation, such as an increasing proportion of patients with neurological deficits or intramural hematoma, may affect risk calculation, even when overt hemodynamic instability is absent. Taken together, these factors reconcile the observation of improved preoperative stability with persistently high predicted operative risk. Importantly, despite this increase in calculated risk, early mortality remained stable and relatively low, underscoring the effectiveness of centralized care, standardized protocols, and high-volume surgical expertise in mitigating the impact of baseline risk on outcomes.

From a technical standpoint, operative strategies remained largely consistent over time, with stable aortic cross-clamp durations and a predominant use of axillary cannulation, in line with previous reports from authoritative authors and international registries such as IRAD [7]. However, cardiopulmonary bypass times significantly increased, reflecting a rise in the proportion of patients undergoing total aortic arch replacement. Importantly, early mortality and major postoperative complications did not significantly change over the fifteen years, remaining consistently low compared with historical benchmarks. Our in-hospital mortality of approximately 11% is notably favorable relative to rates of 18–25% reported in contemporary international series [1,3,8,9]. This stability underscores the maturity of surgical expertise and perioperative care within our regional aortic program, emphasizing the value of a centralized, high-volume referral system in optimizing outcomes.

### 4.2. Sex-Based Analysis: Evolving Profiles and Equivalent Outcomes

Although male predominance persisted, the proportion of female patients undergoing surgery for ATAAD increased progressively over time, reaching 44% in the most recent triennium. This pattern aligns with emerging registry data suggesting a gradual rise in ATAAD incidence among women, potentially reflecting population aging, improved diagnostic awareness, and a greater prevalence of hypertension and degenerative aortic disease in elderly females [5,8,10,11]. In our cohort, women were significantly older and presented with higher EuroSCORE II values, lower body mass index, and better renal function at baseline. They also exhibited a greater prevalence of intramural hematoma—an established presentation in elderly hypertensive females and often associated with less extensive aortic involvement and less frequent propagation of the intimal tear [5,8,12]. Lawrence et al. reported that women generally present with a greater curvature of the aortic sinus wall, which may confer a degree of biomechanical protection against dissection. This anatomical characteristic could partially account for the older age at presentation observed in females with ATAAD [13]. Consistent with this anatomical profile, cross-clamp times were shorter, and total arch replacement was less commonly performed in women. These differences may reflect both intrinsic anatomical factors (smaller aortic diameters, increased vessel fragility, higher risk of bleeding during extensive arch manipulation) and decision-making tendencies toward conservative repair when tissue quality appears unfavorable or comorbidity burden is high [5,8,14,15]. Notably, similar sex-related variations in surgical aggressiveness have been documented in IRAD and other multicenter cohorts, with women more frequently treated with ascending/hemiarch replacement alone and men undergoing more extensive arch procedures [5,8,14,15,16].

Despite these anatomical and procedural distinctions, postoperative outcomes were remarkably similar between sexes. The incidence of major postoperative complications—including stroke, re-exploration for bleeding, and atrial fibrillation—did not differ significantly. However, as previously reported by Norton et al., women in our series required blood transfusion more frequently, which may be explained by smaller circulating blood volume, lower preoperative hemoglobin levels, and greater hemodilution during cardiopulmonary bypass, as also described in sex-specific perfusion physiology literature [16]. Conversely, men experienced a slightly higher rate of postoperative renal injury and dialysis, consistent with their worse preoperative renal function and with prior evidence linking male sex to increased susceptibility to perioperative AKI [8,15].

Short-term mortality was identical in both sexes (10.9% for men and women; *p* = 1.000). This result contrasts with several multicenter studies reporting higher operative mortality in women [5,8,17], yet aligns with other contemporary analyses showing no independent effect of sex on outcomes or even a potential survival advantage for females in selected cohorts [11,13,18]. A recent meta-analysis by Fialka et al. demonstrated that early postoperative mortality in ATAAD is comparable between women and men, with no significant sex-related differences when data are aggregated across available studies [19]. These results indicate that, despite differences in baseline characteristics, comorbidity burden, or surgical approach, short-term survival tends to equalize once patients undergo operative treatment. This supports the hypothesis that early outcomes are driven primarily by preoperative physiological condition and perioperative management rather than by sex itself. Consistent with this, Fukui et al. [20] reported similar findings in a Japanese cohort, with no significant difference in operative mortality between men and women (4.5% vs. 5.8%; *p* = 0.6463) and comparable preoperative risk profiles. Chemtob et al., within the Nordic Consortium for Acute Type A Aortic Dissection, analyzed 1154 surgically treated ATAAD patients across eight Nordic centers, of whom 373 (32%) were female, and, similarly to our experience, women were significantly older at presentation. Hypothermic cardiac arrest time and total operative duration were shorter in females, reflecting a pattern consistent with a more conservative surgical approach [21]. Importantly, no difference was observed in intraoperative mortality (9.1% vs. 6.7%, *p* = 0.17) or 30-day mortality (17.7% vs. 17.4%, *p* = 0.99), and multivariable adjustment confirmed that sex was not an independent determinant of early survival. These results closely mirror our findings. As in our multivariable model, sex (Figure 4) was likewise not associated with in-hospital mortality, supporting the concept that outcome parity between men and women is achievable within standardized, high-volume aortic programs.

In our view, the parity observed in this experience may reflect equitable access to emergent surgical treatment, standardized management pathways within a centralized referral system, and uniform surgical expertise over time—factors that may mitigate historically reported sex disparities and contribute to outcome convergence in modern specialized care.

### 4.3. Implications and Future Perspectives

Our data highlight the critical importance of regionalized aortic care networks that ensure prompt diagnosis, rapid inter-hospital transfer, and the availability of dedicated surgical teams. Such organizational models appear capable of delivering consistently high-quality outcomes across diverse patient profiles, including traditionally higher-risk subgroups such as elderly women. At the same time, ongoing regional epidemiological surveillance is essential for capturing the evolving dynamics of acute aortic syndromes and guiding healthcare planning. While international registries provide essential large-scale data, regional analyses offer valuable insights into context-specific factors—such as referral organization, demographic shifts, and surgical practice patterns—that are often obscured in multicenter datasets. Continuous monitoring at the local level enables healthcare systems to detect emerging trends, including shifts in sex distribution, aging of the treated population, and changing complication profiles. The integration of regional data into national registries and quality improvement programs may ultimately help refine triage strategies, optimize treatment pathways, and improve outcomes for patients with acute aortic syndromes across Italy and Europe.

### 4.4. Limitations

The retrospective design represents the primary limitation of this study. Although the regional database ensured consistent and prospectively maintained data collection, residual confounding and missing variables cannot be entirely excluded. Selection bias is possible, as only patients undergoing surgical repair were included, while medically managed or inoperable cases were not captured. Detailed characterization of malperfusion syndromes was incomplete, limiting precise assessment of organ-specific complications at presentation. Long-term outcomes beyond the early postoperative period were not systematically available, preventing evaluation of late survival and morbidity. Additionally, minor evolutions in institutional practice and perioperative care over the 15-year period may have influenced temporal trends. The COVID-19 pandemic (2019–2021) likely affected surgical volume, potentially impacting observed trends. Seasonal analyses were performed without population-level denominators, limiting precise assessment of incidence variation across calendar periods. Despite these limitations, the study benefits from a large sample size, a homogeneous regional setting with standardized referral and treatment pathways, and an extended observation period, providing a robust representation of real-world surgical practice in ATAAD.

## 5. Conclusions

Over fifteen years of observation, the surgical case volume of ATAAD/IMH in this Northern Italian region remained overall stable. Male predominance persisted, but a non-significant yet progressive increase in female representation was observed. Preoperative shock and intubation became less frequent, suggesting improvements in early diagnostic and referral pathways, while higher predicted risk at admission was not accompanied by increased mortality. Despite older age and higher predicted risk, female patients achieved outcomes comparable to males, in contrast with previous reports from international registries. Importantly, these findings apply exclusively to patients who reached surgery and should not be generalized to those managed conservatively or to long-term survival outcomes. These findings apply exclusively to patients who underwent surgery and cannot be generalized to all acute aortic syndromes or to long-term survival. Nonetheless, results support the value of a structured regional care network, timely referral, and standardized operative strategies in maintaining equitable outcomes across patient subgroups.

## Figures and Tables

**Table 1:** Baseline patient characteristics by period.

	2010–2012	2013–2015	2016–2018	2019–2021	2022–2024	*p*	*p* for Trend	Trend
*n*	75	72	104	72	104			
Age, median (IQR)	66 (60, 75)	66 (59, 73)	70 (63, 76)	68 (58, 74)	71 (59, 77)	0.100	0.064	↑
Age class, *n* (%)						0.139	0.105	↑
<55	14 (18.7)	13 (18.1)	12 (11.5)	8 (11.1)	17 (16.3)			
55–74	41 (54.7)	43 (59.7)	56 (53.8)	48 (66.7)	48 (46.2)			
75+	20 (26.7)	16 (22.2)	36 (34.6)	16 (22.2)	39 (37.5)			
Female, *n* (%)	22 (29.3)	25 (34.7)	36 (34.6)	21 (29.2)	46 (44.2)	0.208	0.074	↑
BMI, median (IQR)	26.7 (24.8, 29.3)	26.7 (24.4, 30.7)	27.7 (25.3, 30.5)	26.2 (24.0, 28.4)	26.1 (23.1, 30.8)	0.412	0.265	-
Creatinine pre, median (IQR) (7% missing)	1.00 (0.81, 1.09)	1.00 (0.90, 1.19)	1.00 (0.86, 1.28)	1.01 (0.84, 1.27)	1.00 (0.90, 1.19)	0.855	0.595	-
Dialisys pre, *n* (%)	1 (1.3)	1 (1.4)	0 (0.0)	0 (0.0)	1 (1.0)	0.816	0.394	-
Redo, *n* (%)	4 (5.3)	3 (4.2)	6 (5.8)	2 (2.8)	5 (4.8)	0.937	0.655	-
Diabetes, *n* (%)	6 (8.0)	3 (4.2)	6 (5.8)	3 (4.2)	6 (5.8)	0.861	0.694	-
Neurological deficit, *n* (%) (5% missing)	4 (6.2)	2 (3.3)	9 (8.7)	4 (5.6)	13 (12.5)	0.269	0.102	↑
LVEF, median (IQR) (4% missing)	60 (50, 60)	55 (50, 60)	55 (55, 60)	57 (55, 60)	55 (55, 60)	0.118	0.198	-
Intubation pre, *n* (%)	25 (33.3)	18 (25.0)	14 (13.5)	9 (12.5)	14 (13.5)	0.002	0.021	↓
Shock, *n* (%)	24 (32.0)	20 (27.8)	9 (8.7)	7 (9.7)	9 (8.7)	<0.001	<0.001	↓
Log EuroSCORE, median (IQR) (4% missing)	30.9 (19.7, 44.9)	30.1 (19.7, 45.5)	31.6 (20.2, 46.3)	27.3 (16.1, 40.7)	34.4 (20.5, 52.4)	0.140	0.245	-
EuroSCORE II, median (IQR) (37% missing)	/	/	14.1 (7.3, 23.5)	9.1 (5.4, 18.5)	15.5 (8.6, 24.2)	0.002	0.085	↑
GERAADA score, median (IQR)	13.1 (10.0, 19.3)	14.1 (10.5, 20.9)	11.3 (9.6, 15.3)	11.8 (9.3, 14.2)	13.9 (10.7, 20.1)	0.002	0.324	-

Trends with *p* < 0.2: ↓ decreasing trend; ↑ increasing trend.

**Table 2:** Operative details by study period.

	2010–2012	2013–2015	2016–2018	2019–2021	2022–2024	*p*	*p* for Trend	Trend
*n*	75	72	104	72	104			
Cannulation axillary artery, *n* (%)	43 (57.3)	48 (66.7)	65 (62.5)	47 (65.3)	64 (61.5)	0.802	0.505	-
Cross-clamp minutes, median (IQR)	93 (64, 135)	93 (71, 121)	90 (63, 122)	105 (80, 156)	103 (76, 143)	0.088	0.061	↑
CPB minutes, median (IQR)	137 (99, 185)	127 (103, 178)	134 (103, 183)	169 (135, 241)	175 (138, 224)	<0.001	<0.001	↑
Root surgery, *n* (%)	19 (25.3)	16 (22.2)	15 (14.4)	17 (23.6)	14 (13.5)	0.132	0.065	↓
Total arch replacement, *n* (%)	12 (16.0)	9 (12.5)	18 (17.3)	19 (26.4)	32 (30.8)	0.017	0.002	↑

Trends with *p* < 0.2: ↓ decreasing trend; ↑ increasing trend.

**Table 3:** Outcomes by study period.

	2010–2012	2013–2015	2016–2018	2019–2021	2022–2024	*p*	*p* for Trend	Trend
*n*	75	72	104	72	104			
ICU days, median (IQR) (2% missing)	5 (3, 11)	4 (3, 10)	5 (3, 10)	6 (3, 9)	4 (3, 10)	0.826	0.679	-
Red blood cell units, median (IQR) (8% missing)	7 (3, 12)	4 (2, 7)	2 (2, 4)	4 (2, 6)	4 (2, 6)	<0.001	0.094	↓
Platelet and Plasma units, median (IQR) (36% missing)	3 (2, 5)	2 (1, 4)	1 (0, 3)	0 (0, 0.50)	3 (2, 3)	<0.001	0.004	↓
Stroke (%)	11 (14.7)	9 (12.5)	7 (6.8)	8 (11.1)	10 (9.6)	0.489	0.378	-
Resternotomy for bleeding (%) (5% missing)	11 (14.7)	8 (11.1)	11 (10.7)	11 (15.3)	13 (12.5)	0.870	0.879	-
Resternotomy for Hemodynamic Instability (%)	1 (1.5)	2 (3.4)	3 (2.9)	3 (4.2)	3 (2.9)	0.928	0.617	-
Creatinine at 72 h, median (IQR) (73% missing)	/	1.00 (1.00, 1.00)	1.38 (1.08, 2.42)	1.33 (1.08, 2.10)	1.50 (0.96, 2.05)	0.742	0.443	-
Dialysis post (%)	4 (5.3)	5 (6.9)	19 (18.3)	8 (11.1)	11 (10.6)	0.068	0.172	-
Abdominal Complication Requiring Surgery (%) (5% missing)	0 (0.0)	0 (0.0)	0 (0.0)	1 (1.4)	2 (1.9)	0.462	0.096	↑
Atrial Fibrillation (%)	27 (36.0)	26 (36.1)	45 (43.3)	21 (29.2)	45 (43.3)	0.285	0.399	-
Intraoperative mortality, *n* (%)	1 (1.3)	2 (2.8)	1 (1.0)	1 (1.4)	4 (3.9)	0.688	0.458	-
In-hospital mortality, *n* (%)	9 (12.0)	6 (8.3)	8 (7.8)	8 (11.1)	15 (14.6)	0.561	0.572	-

Trends with *p* < 0.2: ↓ decreasing trend; ↑ increasing trend.

**Table 4:** Baseline patient characteristics based on sex.

	Male	Female	*p*
*n*	277	150	
Intramural hematoma, *n* (%)	3 (1.1)	8 (5.3)	0.020
Age, median (IQR)	66 (56, 73)	73.50 (65, 78)	<0.001
Age class, *n* (%)			<0.001
<55	56 (20.2)	8 (5.3)	
55–74	163 (58.8)	73 (48.7)	
75+	58 (20.9)	69 (46.0)	
BMI, median (IQR)	27.7 (24.9, 30.8)	25.1 (22.8, 28.3)	<0.001
Creatinine pre, median (IQR)	1.03 (0.94, 1.32)	0.90 (0.70, 1.02)	<0.001
Dialisys pre, *n* (%)	2 (0.7)	1 (0.7)	1.000
Redo, *n* (%)	12 (4.3)	8 (5.3)	0.638
Diabetes, *n* (%)	13 (4.7)	11 (7.3)	0.276
Neurological deficit, *n* (%)	20 (7.7)	12 (8.3)	0.849
LVEF, median (IQR)	55 (55, 60)	55 (55, 60)	0.147
Intubation pre, *n* (%)	56 (20.2)	24 (16.0)	0.302
Shock, *n* (%)	48 (17.3)	21 (14.0)	0.411
Log EuroSCORE, median (IQR)	25.2 (19.5, 40.7)	41.6 (28.7, 56.8)	<0.001
EuroSCORE II, median (IQR)	11.9 (6.0, 20.4)	16.0 (9.2, 26.2)	0.002

**Table 5:** Intraoperative characteristics and outcomes based on sex.

	Male	Female	*p*
*n*	277	150	
Cannulation axillary artery, *n* (%)	175 (63.2)	92 (61.3)	0.754
Cross-clamp minutes, median (IQR)	103 (73, 146)	88 (68, 111)	<0.001
CPB minutes, median (IQR)	157 (114, 212)	145 (109, 179)	0.025
Root surgery, *n* (%)	58 (20.9)	23 (15.3)	0.196
Replacement arch, *n* (%)	69 (24.9)	21 (14.0)	0.009
ICU days, median (IQR)	5 (3, 11)	5 (3, 8)	0.909
Red blood cell units, median (IQR)	3 (2, 6)	4.50 (2, 7)	0.013
Platelet and Plasma units, median (IQR)	3 (1, 4)	3 (1, 3)	0.744
Stroke (%)	25 (9.0)	20 (13.4)	0.186
Resternotomy for bleeding (%)	39 (14.1)	15 (10.1)	0.286
Resternotomy for Hemodynamic Instability (%)	8 (3.0)	4 (2.8)	1.000
Creatinine at 72 h, median (IQR)	1.64 (1.18, 2.44)	1.21 (0.98, 1.55)	0.002
Dialysis post (%)	38 (13.7)	9 (6.0)	0.015
Abdominal Complication Requiring Surgery (%)	2 (0.8)	1 (0.7)	1.000
Atrial Fibrillation (%)	100 (36.1)	64 (42.7)	0.211
Intraoperative mortality, *n* (%)	3 (1.1)	6 (4.0)	0.072
In-hospital mortality, *n* (%)	30 (10.9)	16 (10.7)	1.000

## Data Availability

The data presented in this study are available on request from the corresponding author. The data are not publicly available due to data protection directive 95/46/EC.

## Abbreviations

The following abbreviations are used in this manuscript:

AAD	Acute aortic dissection
ATAAD	Acute type A aortic dissection
AUC	Area Under the Curve
BMI	Body Mass Index
CI	Confidence Interval
CPB	Cardiopulmonary Bypass
IMH	intramural hematoma
IQR	Interquartile Range
IRAD	International Registry of Acute Aortic Dissection
OR	Odds Ratio
PAU	Penetrating Atherosclerotic Ulcer
